# Numerical Investigation into In-Plane Crushing of Tube-Reinforced Damaged 5052 Aerospace Grade Aluminum Alloy Honeycomb Panels

**DOI:** 10.3390/ma14174992

**Published:** 2021-09-01

**Authors:** Younes Djemaoune, Branimir Krstic, Stefan Rasic, Daniel Radulovic, Marjan Dodic

**Affiliations:** 1 Section of Aerospace Engineering and Mechanics, Department of Military Mechanical Engineering, Military Academy, University of Defence in Belgrade, Military Academy, 11042 Belgrade, Serbia; younes.djemaoune@gmail.com (Y.D.); rasha.soad@gmail.com (S.R.); radulovic1995@gmail.com (D.R.); marjan.dodic@va.mod.gov.rs (M.D.); 2 Department of Aerospace Engineering, Military Technical Institute, 11030 Belgrade, Serbia

**Keywords:** aluminum honeycomb, in-plane compression, finite element analysis, energy absorption, crashworthiness

## Abstract

This paper aims to investigate the crashworthiness performance degradation of a damaged 5052 aluminum honeycomb panels under in-plane uniaxial quasi-static compression and the possibility of improving it using reinforcement tubes. The in-plane crushing behaviors and energy absorption capacities of the intact, damaged, and tube-reinforced damaged panels with different damage sizes in both X_1_ and X_2_ directions are numerically simulated by using the nonlinear FE method Abaqus/Explicit, and the crashworthiness performances are compared with each other. The validation of finite element model involves comparing the obtained simulation results with theoretical and experimental ones. Very good agreement between numerical, experimental, and theoretical results is achieved. The first maximum compressive load and the mean crushing load of the different honeycomb configurations are analyzed and compared through the load–strain curves. The energy absorption capacity of the damaged and the tube-reinforced damaged panels is calculated and then compared with their corresponding intact ones. The deformation modes are explained in detail. The obtained results show that the crashworthiness performance degradation is directly proportional to the damage size as well as the insertion of reinforcement tubes considerably improves in-plane crushing resistance of damaged honeycomb panels.

## 1. Introduction

**Figure 1:**
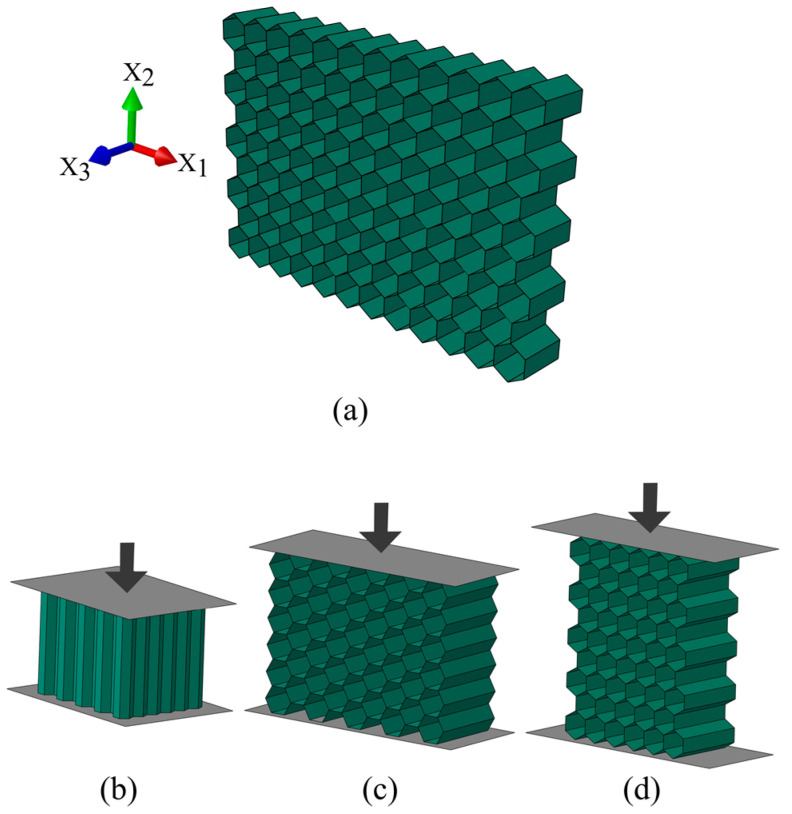
(**a**) Schematic of hexagonal honeycomb; (**b**) out-of-plane compression; (**c**) in-plane compression in the X_1_ direction; (**d**) in-plane compression in the X_2_ direction.

Honeycomb structures are attractive candidates to be widely used in lightweight engineering design and energy absorbing applications, especially in the aerospace and automotive industries, due to their high stiffness-to-weight ratio, high strength-to-weight ratio, cost efficiency, multifunctionality, and extraordinary energy absorption capacity [1,2,3,4,5,6,7,8,9,10]. Mechanical behavior of honeycomb structure (Figure 1a) under quasi-static and dynamic compressive loading have been intensively studied, experimentally and numerically, in the out-of-plane direction (Figure 1b) [11,12,13,14,15] as well as in the in-plane direction (Figure 1c,d) [16,17,18,19,20,21,22,23]. Lorna J. Gibson and Michael F. Ashby [1] investigated the mechanical behavior of honeycomb structure in both out-of-plane and in-plane directions by detailed studying the micromechanics of the single cell.

The 5052 expanded aerospace grade aluminum honeycomb materials are predominantly used in sandwich structures to meet design requirements for highly engineered structural components. As a structural core material, it finds applications in all types of aerospace vehicles and supporting equipment where sandwich structures offer rigid panels of minimum weight, aerodynamic smooth surfaces, and high fatigue resistance [24]. Consequently, a large number of authors test and investigate the characteristics and mechanical behavior of the 5052 aluminum honeycombs under various types of loading [25,26,27,28].

In order to improve the mechanical properties of honeycomb structure and its energy absorption capacity, a novel design consisted of honeycomb panel filled with tubes or foam was recently developed. Numerous investigations have been carried out concerning this topic and important results have already been reached [29,30,31,32,33,34,35,36]. Zhang et al. [29] studied the quasi-static compressive of tube-reinforced aluminum honeycomb. Compared with empty honeycomb panel, the ones filled with tubes shown significant improvements in the mechanical properties and energy absorption capacity. The in-plane compressive response of the foam filled aluminum has been investigated experimentally and numerically by Mozafari et al. [33]. It was concluded that the foam filling of honeycomb core can increase the in-plane crushing strength up to 208 times and its specific absorbed energy up to 20 times.

Honeycomb core sandwich panels are susceptible to damage from impact events such as bird strike, dropped tools during aircraft maintenance and tarmac debris kicked-up by the aircraft wheels during take-off or landing can damage the panel and cause substantial degradation of mechanical properties of the structure. Ajdari et al. [37] employed finite element method (FEM) to study the role of irregularities, in the form of a missing cell cluster and variations in the cell arrangements, on the energy absorption under different crushing velocities. The results suggest that the energy absorption capacity of the honeycomb structure with a lower relative density are more sensitive to the presence of a defect. Zhang et al. [38] found that the compressive strength of the open-hole damaged honeycomb may decline about 66% of that of the intact plate due to the stress concentration at the equators of the hole and the local buckling of the honeycomb. After the repair process, the strength of the repaired plate could resume to 76% of that of the intact plate.

In some applications, such as using an aluminum honeycomb panel as an energy absorber, the panel could be damaged, and an appropriate reparation is immediately re-quired. In the present study, a solution using reinforcement tube is proposed to reinforce the damaged panels in order to recover the mechanical properties of the damaged panel and restore its energy absorption capacity. For that reason, the responses of damaged and tube-reinforced damaged panels under in-plane quasi-static compressive loading are investigated numerically using the finite element code Abaqus/Explicit. The validation of the finite element (FE) model of intact panel involves comparing simulation results with theoretical values and experimental results published by Papka and Kyriakides [17,18]. The damaged panels are modeled from the intact ones by cutting the centered hole whose area represents 5%, 10%, or 20% of the total area of the intact panel. To reinforce the damaged panels, the tubes with three different wall thicknesses are inserted exactly in the cut hole. The numerical simulation of the mechanical behavior of the intact, damaged, and tube-reinforced damaged panel was performed under the same conditions. In order to investigate the level of property degradation of damaged honeycomb panels and the possibility of improving it using reinforcement tubes, the first maximum compressive load, the mean crushing load and the absorbed energy capacity as well as the deformation mode of these panels were analyzed and compared to their corresponding intact ones.

## 2. In-Plane Properties of Honeycomb under Uniaxial Compressive Loading

**Figure 2:**
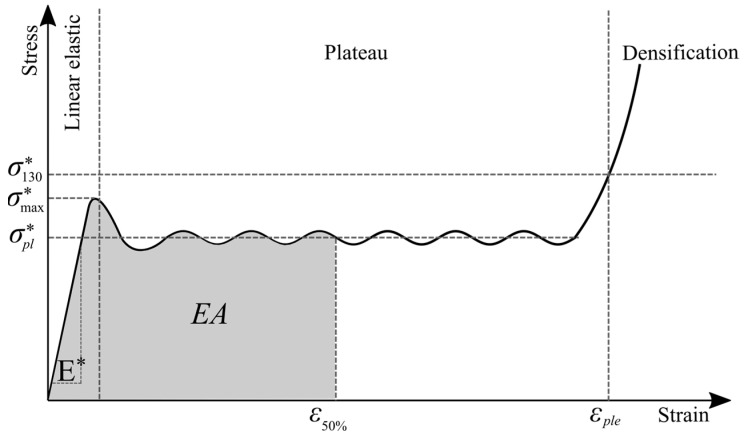
Typical stress–strain curve of an elastic- plastic honeycomb under in-plane uniaxial compression.

Typical stress–strain curve of an elastic–plastic honeycomb under in-plane uniaxial compression shows three distinct regimes of behavior: linear–elastic, plateau, and densification (Figure 2).

Each of these regimes of behavior is associated with the mechanism by which the cells deform within that regime [39]. The linear–elastic behavior of honeycomb within the first regime (Figure 2) is produced by bending in the cell walls due to relatively low initial loads. The appropriate linear–elastic response is determined by set of five moduli, which can be calculated as follows [1]:

(1) $$E_{1}^{*}=E_{s}{\left(\frac{t}{l}\right)}^{3}\frac{\cos\theta }{\left(\frac{h}{l}+\sin\theta \right)\sin^{2}\theta },$$

(2) $$E_{2}^{*}=E_{s}{\left(\frac{t}{l}\right)}^{3}\frac{\left(\frac{h}{l}+\sin\theta \right)}{\cos^{3}\theta },$$

(3) $$\nu_{12}^{*}=\frac{\cos^{2}\theta }{\left(\frac{h}{l}+\sin\theta \right)\sin\theta },$$

(4) $$\nu_{21}^{*}=\frac{\left(\frac{h}{l}+\sin\theta \right)\sin\theta }{\cos^{2}\theta },$$

(5) $$G_{12}^{*}=E_{s}{\left(\frac{t}{l}\right)}^{3}\frac{\left(\frac{h}{l}+\sin\theta \right)}{{\left(\frac{h}{l}\right)}^{2}\left(1+\frac{16h}{l}\right)\cos\theta }$$

Plastic collapse of cells occurs when the bending moment in the cell walls reaches the fully plastic moment giving a stress–strain curve with a horizontal plateau (Figure 2). The plateau stresses in the X_1_ and X_2_ directions corresponding to plastic yielding [1]:

(6) $${\left(\sigma_{pl}^{*}\right)}_{1}=\sigma_{ys}{\left(\frac{t}{l}\right)}^{2}\frac{1}{2\left(\frac{h}{l}+\sin\theta \right)\sin\theta },$$

(7) $${\left(\sigma_{pl}^{*}\right)}_{2}=\sigma_{ys}{\left(\frac{t}{l}\right)}^{2}\frac{1}{2\cos^{2}\theta }$$

In the end, at relatively large compressive strains (typically about 0.8), the opposing cell walls begin to meet and touch (or their broken fragments pack together) and further deformation compresses the cell wall material itself [1]. As the material densifies, the stress rises steeply giving the final portion of the stress–strain curve marked as densification in Figure 2. In case of regular hexagonal cells (*θ* = 30°; *h = l*), the honeycomb will have the same properties in both X_1_ and X_2_ directions and the abovementioned equations can be simplified [1]:

(8) $$E^{*}=E_{1}^{*}=E_{2}^{*}=2.3E_{s}{\left(\frac{t}{l}\right)}^{3},$$

(9) $$\nu^{*}=\nu_{12}^{*}=\nu_{21}^{*}=1,$$

(10) $$\sigma_{pl}^{*}={\left(\sigma_{pl}^{*}\right)}_{1}={\left(\sigma_{pl}^{*}\right)}_{2}=\frac{2}{3}\sigma_{ys}{\left(\frac{t}{l}\right)}^{2}$$

The energy absorption of honeycomb structure is defined as the area under the stress–strain curve up to 50% strain [40], Figure 2 and can be expressed as:

(11) $$EA=\frac{L^{0}}{100}\int_{0}^{\varepsilon_{50\%}}Fd\varepsilon$$

In addition to above equations, Young’s modulus E*, plateau stress $\sigma_{pl}^{*}$ and energy absorption *EA* can also be determined from the stress–strain curve [40] created during experimental tests or numerical simulations as shown in Figure 2.

## 3. Finite Element Models

**Figure 3:**
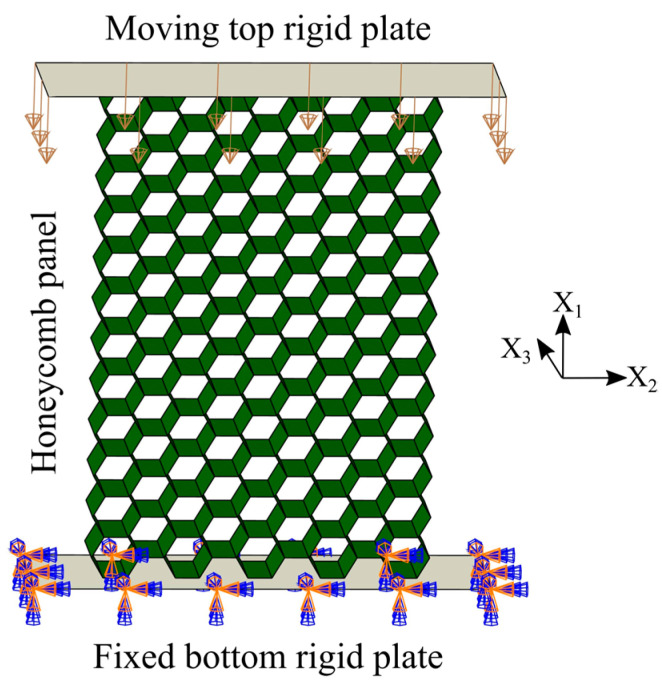
FE model elements (intact panel under compression in the X_1_ direction).

In order to predict the mechanical behavior of damaged and tube-reinforced damaged honeycomb panels under uniaxial quasi-static compression in the X_1_ and the X_2_ directions, the appropriate finite element models are developed in Abaqus/Explicit. The models assume that the honeycomb panel (intact, damaged, and tube-reinforced damaged) is placed on the completely fixed bottom rigid plate and compressed in X_1_ or X_2_ direction by moving top rigid plate at constant velocity, Figure 3.

**Figure 4:**
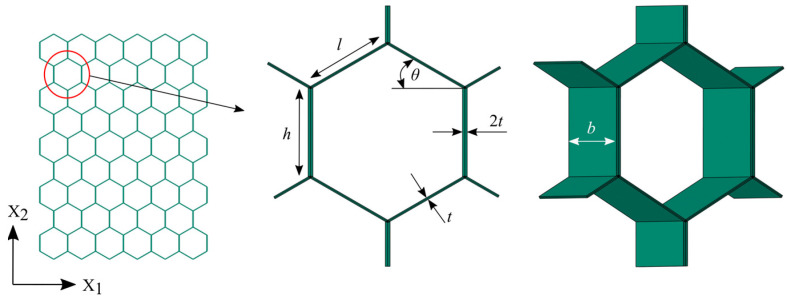
Honeycomb hexagonal cell geometric parameters.

The honeycomb structure is designed as a set of regular hexagonal cells (*l* = *h* = 5.5 mm; *t =* 0.145 mm; *b =* 0.8 mm; *θ*= 30°), Figure 4. Reinforcement tubes are modeled with six different radiuses and three different wall thicknesses *e* (*t*; *2t*; *4t*). The cell wall and reinforcement tubes material is aerospace grade aluminum alloy Al-5052-H39 and considered in the FE models as an isotropic, elastic–plastic material with following mechanical properties [18]: density *ρ* = 2.68 g/cm^3^, Young’s modulus *E*_*s*_ = 68.97 GPa, Poisson’s ratio *ν* = 0.33, and static yield strength σ_*ys*_ = 292 MPa. Moreover, its behavior is described using bilinear isotropic hardening model with tangent modulus *E*_tan_ = *E*_*s*_/100 = 0.6897 GPa [18].

To achieve quasi-static conditions, the velocity of moving plate is set to *V =* 1 mm/s [40]. The contact between the honeycomb panels, the rigid plates and the reinforcement tubes is specified as explicit surface interaction with frictional coefficient *f =* 0.2 [18]. In order to avoid the global buckling, all nodes of the FE model of honeycomb panels, reinforcement tubes and rigid plates are constrained to move vertically in the X_1_–X_2_ plane.

**Figure 5:**
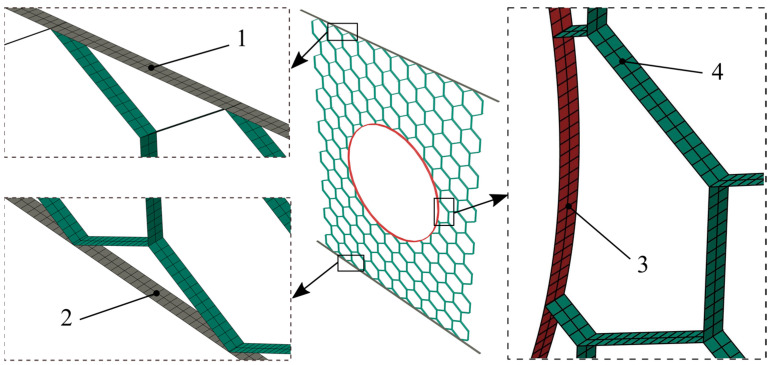
FE model elements and meshes: 1—moving top rigid plate, 2—fixed bottom rigid plate, 3—reinforcement tube, 4—honeycomb panel.

In the presented FE model, the honeycomb panel and reinforcement tubes are discretized with a 4-node, quadrilateral, stress/displacement shell element S4R with reduced integration and a large-strain formulation, Figure 5. Furthermore, the top and bottom rigid plates are meshed using 4-node three-dimensional bilinear rigid quadrilateral element R3D4, Figure 5. The selected mesh size of 0.40 mm for all components provides high accuracy of calculation. Finally, in order to reduce the cost of the analysis, an appropriate mass scaling method is applied by scaling the mass of the elements with stable time increments less than the time target value fixed to 10^−5^ s. The ratios of kinetic energies (ALLKE) to their correspondent internal energies (ALLIE), for all simulations, were checked to supervise the precision of the results. The mesh parameters are summarized in Table 1.

### 3.1. Intact Panel Models

**Figure 6:**
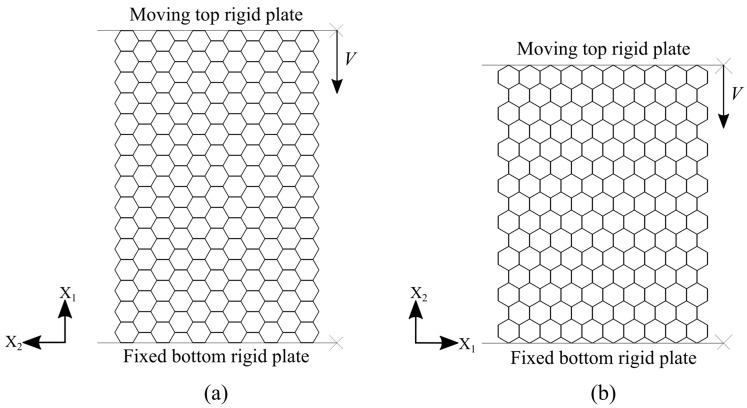
Intact panel models: (**a**) compression in the X_1_ direction; (**b**) compression in the X_2_ direction.

The intact honeycomb panels are dimensioned in accordance with the International Standard Mechanical testing of metals—ductility testing—compression test for porous and cellular metals [40]. Two types of panels are created. The intact panel for the purposes of the compression simulation in the X_1_ direction consists of 15 × 11 (X_1_ × X_2_) cells i.e., 142.95 mm × 93.54 mm (X_1_ × X_2_), Figure 6a. The one dedicated to the simulation of compressive loading along the X_2_ direction consists of 10 × 15 (X_1_ × X_2_) cells i.e., 95.3 mm × 126.55 mm (X_1_ × X_2_), Figure 6b. Detailed finite element model of intact panel was firstly built in Abaqus/Explicit environment and thereafter validated by theoretical values and experimental results published by Papka [18].

### 3.2. Damaged Panel Models

**Figure 7:**
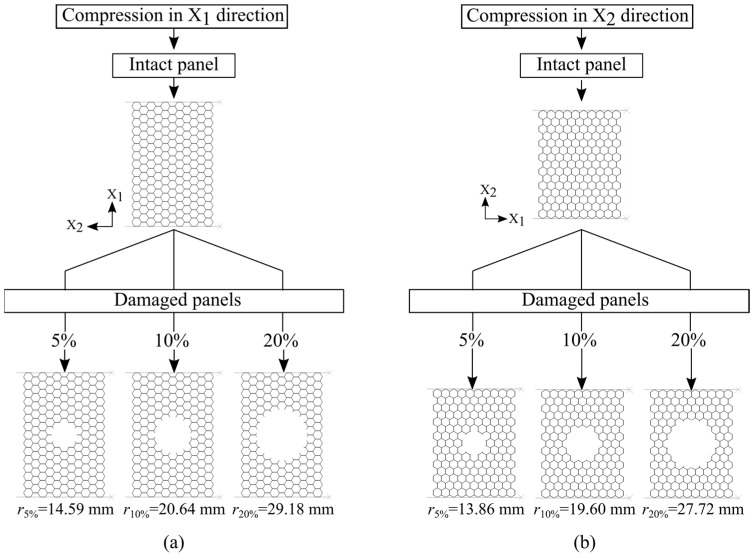
Damage panel models: (**a**) compression in the X_1_ direction; (**b**) compression in the X_2_ direction.

The damaged panels are modeled from the intact ones by cutting the perfect circular hole in the middle of the panels with the radiuses of 14.59 mm, 20.64 mm, and 29.18 mm (compression in the X_1_ direction) and 13.86 mm, 19.60 mm, and 27.72 mm (compression in the X_2_ direction) which represent the loss of 5%, 10%, and 20% of the total honeycomb intact panel area, respectively, Figure 7.

### 3.3. Tube-Reinforced Damaged Panel Models

**Figure 8:**
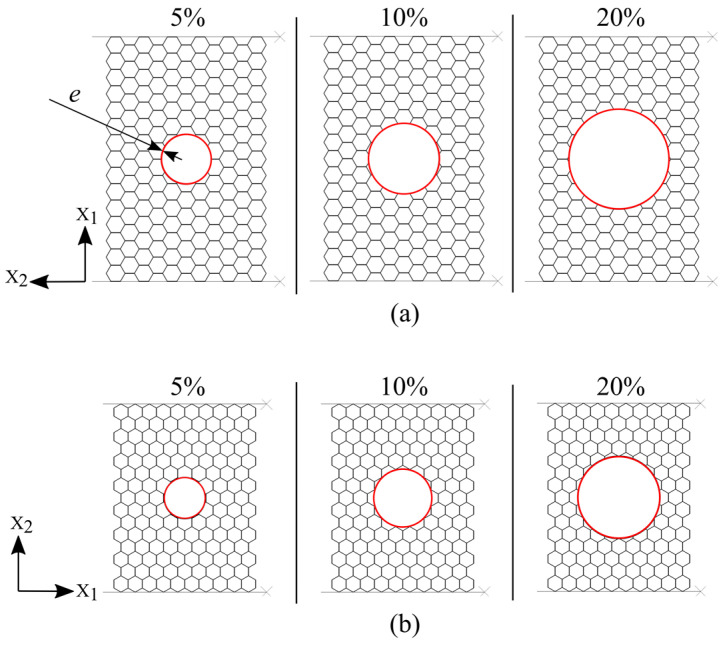
Tube-reinforced damaged panel models: (**a**) compression in the X_1_ direction; (**b**) compression in the X_2_ direction.

According to the damage sizes, the appropriate reinforcement tubes with three different wall thicknesses *e* (*t*; *2t*; *4t*) are inserted exactly in the cut hole of the damaged panels, Figure 8.

## 4. Results and Discussion

### 4.1. Intact Panel Model Validation

**Figure 9:**
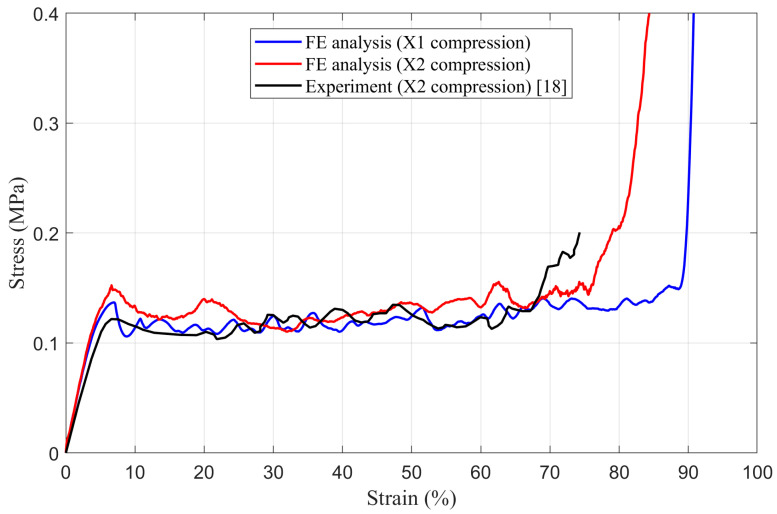
The stress–strain behavior of intact panels.

**Figure 10:**
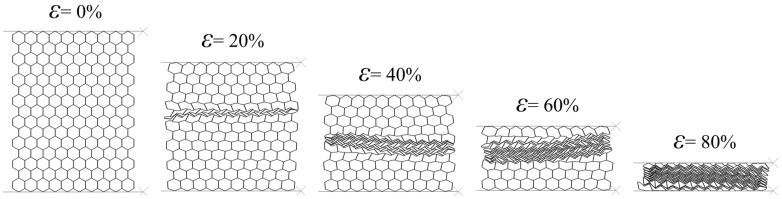
Sequence of deformed configurations of intact panel under compression in the X_2_ direction.

The accuracy of the numerical models of intact panels are validated using theoretical values and experimental results [18]. The stress–strain responses of intact honeycomb panels under uniaxial quasi-static compressive loading in the X_1_ and the X_2_ direction obtained from numerical simulation are presented in Figure 9. When the stress σ* is plotted against the strain ε, the values of Young’s modulus E*, the first maximum compressive strength $\sigma_{\max}^{*}$ (compressive stress corresponding to the first local maximum in the stress–strain curve) and plateau stress $\sigma_{pl}^{*}$ of the honeycomb panel are estimated according to [40] and then compared with theoretical (calculated by Equation (8) and Equation (10)) and experimental ones [18], Table 2. By analyzing the data (Figure 9 and Table 2), it can be observed that the numerical and the theoretical results are in very good agreement. On the other side, the evident mismatch between the experimental and the numerical results is a direct consequence of the presence of imperfections occurred during the honeycomb panel production process (inhomogeneity of a printed adhesive lines, solidification and curing of adhesive, amount of applied pressure and heat up rate, etc.) which decrease Young’s modulus, first maximum compressive stress and plateau stress. It should also be noted that the numerical simulation results present a moderate middle ground between experimental and theoretical ones. Moreover, the numerical simulation of crushing behavior of intact panel under compression in the X_2_ direction shows a horizontal collapse row by row, like an “I” band perpendicular to the loading direction, and fully corresponds to the results published by Papka [18], Figure 10. Based on the aforementioned considerations, it was concluded that the finite element models of intact panel are accurate enough to predict crashworthiness performance of damaged and tube-reinforced damaged honeycomb panels.

### 4.2. Damaged Panel Models

**Figure 11:**
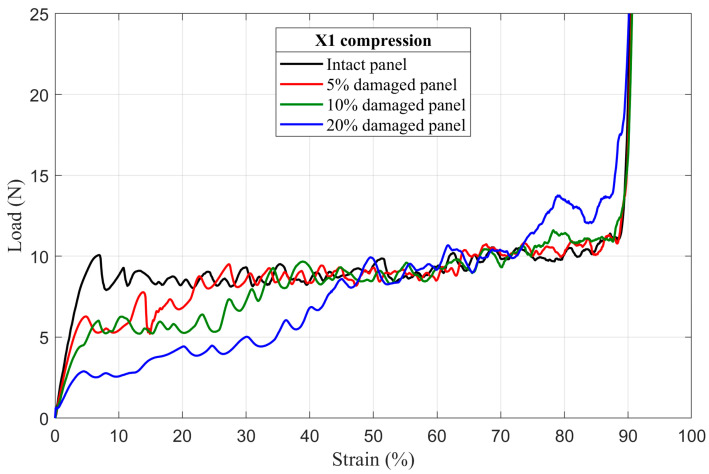
The load–strain behavior of damaged panels under compression in the X_1_ direction.

**Figure 12:**
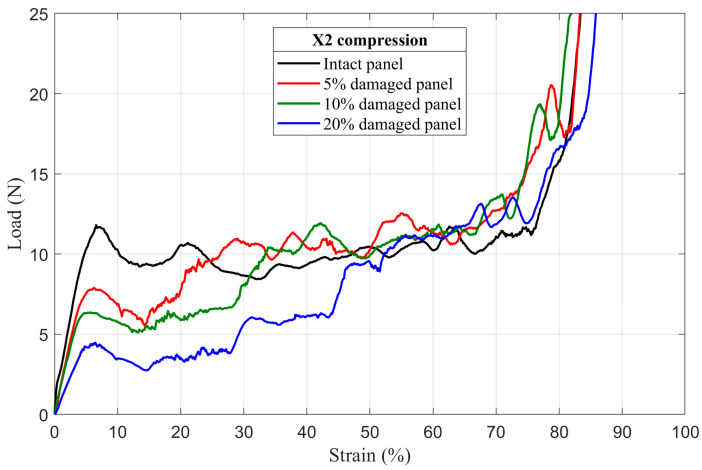
The load–strain behavior of damaged panels under compression in the X_2_ direction.

The results of numerical simulation of mechanical behavior of damaged honeycomb panels under uniaxial quasi-static compressive loading in the X_1_ and the X_2_ directions, are presented in the form of load–strain curves in Figure 11 and Figure 12, respectively. For easier comparison of results, the appropriate intact panel curves were also added into figures.

#### 4.2.1. First Maximum Compressive Load

It is clear from Figure 11 and Figure 12 that the first maximum compressive load decreases as the radius of the circular hole (damage) in the middle of the damaged panels increases. An increase in damage radius results in a decrease in minimum cross section area of the panel. Therefore, the downward trend in the first maximum compressive load is directly related to reducing the number of load-bearing elements (cell walls) contained in the minimum cross section of the damaged honeycomb panels.

#### 4.2.2. Deformation Mode

**Figure 13:**
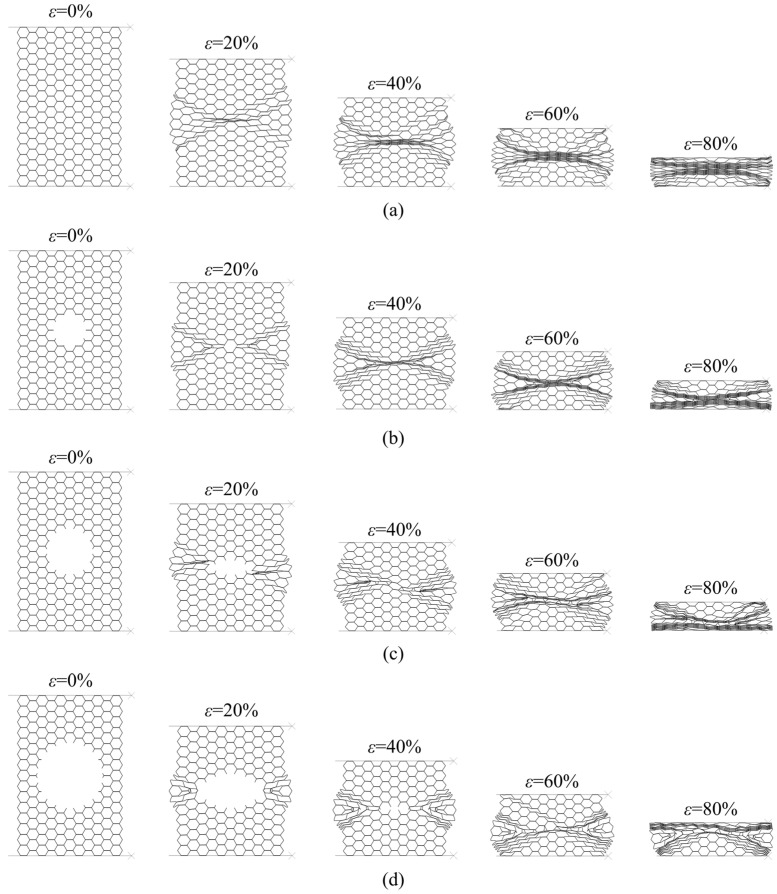
Sequence of deformed configurations under compression in the X_1_ direction: (**a**) intact panel; (**b**) 5% damaged panel; (**c**) 10% damaged panel; (**d**) 20% damaged panel.

**Figure 14:**
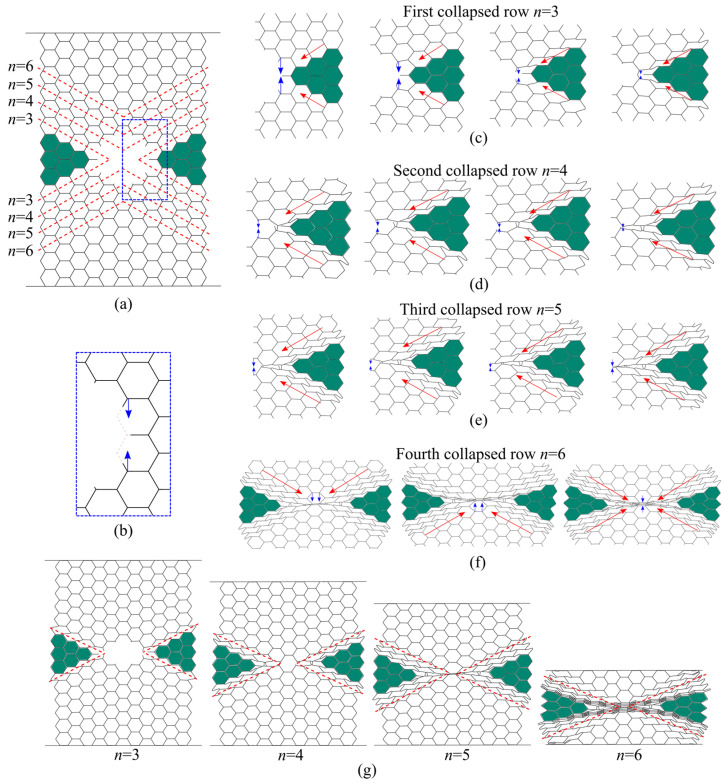
Deformation modes of damaged panels under compression in the X_1_ direction: (**a**) configuration of damaged panel before crushing; (**b**) local crushing of the cut cell walls; (**c**) crushing of the first collapsed row; (**d**) crushing of the second collapsed row; (**e**) crushing of the third collapsed row; (**f**) crushing of the fourth collapsed row; (**g**) sequence of deformed configurations.

In the X_1_ compression direction, double-thickness cell walls are perpendicular to the load. In this case, the load is principally carried by the single-thickness cell walls (inclined walls). Damaged panels, like their intact counterparts, in this loading direction show shear bands with “X” shape due to the bending of the cell walls [1,16,20,25,41,42], Figure 13. For damaged panels, the deformation first occurs locally by crushing of the cut cell walls (incomplete cells without all six walls) which cannot carry the load (Figure 14a,b). This local crush is immediately followed by symmetrical shear of inclined rows with minimum cell number (the first collapsed rows with *n* = 3 cells marked by red arrows in the Figure 14c). Once the first inclined rows fully collapsed, the damage hole decreases and the next row with pseudo-cut cell walls becomes the new weakest zone that cannot support the load. Thus, the inclined row with *n* = 4 cells collapses with the same mechanism along the same shear direction (Figure 14d), and so on until the total closure of the damage hole (rows with *n* = 5 cells in the Figure 14e). After that, the inclined rows, above and below the damage, with *n* = 6 cells shear from the two sides (red arrows in the Figure 14f) and crush the central cells vertically (blue arrows in the Figure 14f). The cells between the first inclined collapsed rows (green colored cells in the Figure 14g) form an unloaded zone which stays intact (or slightly deformed) and move left/right from the damage until the densification occurs.

**Figure 15:**
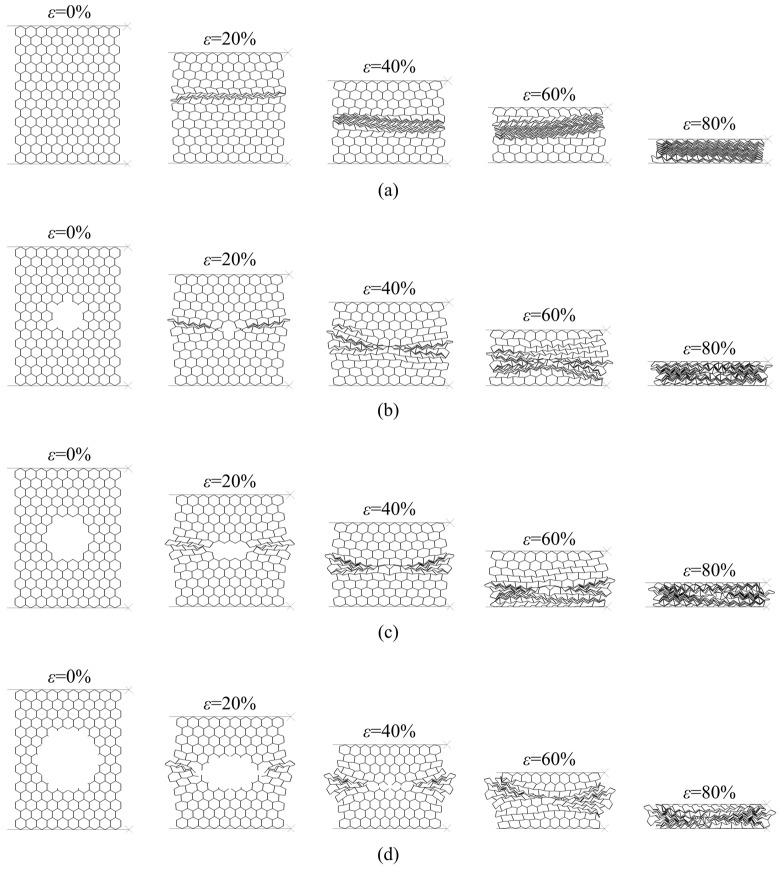
Sequence of deformed configurations under compression in the X_2_ direction: (**a**) intact panel; (**b**) 5% damaged panel; (**c**) 10% damaged panel; (**d**) 20% damaged panel.

**Figure 16:**
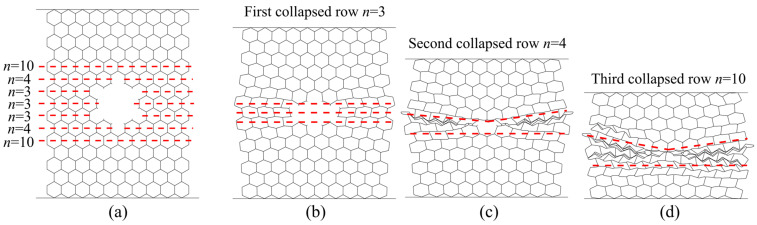
Deformation modes of damaged panels under compression in the X_2_ direction: (**a**) configuration of damaged panel before crushing; (**b**) crushing of the first collapsed row; (**c**) crushing of the second collapsed row; (**d**) crushing of the third collapsed row.

In contrast to the X_1_ direction, double-thickness cell walls are parallel to the load in the case of compression in the X_2_ direction and accordingly they carry the largest amount of the load. Damaged panels show slightly deformed shear bands with “I” shape, Figure 15. The weakest part of the panel is the cross sections with the lowest number of cells i.e., rows with *n* = 3 cells in the Figure 16a. Therefore, these rows collapse first, Figure 16b. This is followed by symmetric collapse of the rows with the first greater cell number (rows with *n* = 4 cells in the Figure 16c). After completely closing the damage hole, the adjacent rows, above and below the damage, collapse successively and symmetrically, one by one, until the densification occurs (Figure 16d).

#### 4.2.3. Mean Crushing Load

As stated in the previous section, after the first rows collapse, the damage hole begins to close and its size becomes smaller and leads to an increase in the number of load-bearing elements, Figure 14 and Figure 16. Consequently, as shown in Figure 11 and Figure 12, it can be observed that, in both loading directions, the plateau regimes of all damaged panels present a staircase increase in load. The number of load steps varies from one damaged panel to another, in function of the number of collapsed rows before the total closing of the damage. Therefore, just after the damage closed and before densification, the damaged panels show approximately the same mean crushing load (the mean load in the plateau regime) values as their intact counterparts with some undulations caused by the non-homogeneity of the panel due to the defects, especially along X_2_ loading direction.

#### 4.2.4. Energy Absorption

This type of structure, as already mentioned in the introduction section, is usually used in energy absorption applications because of the large amount of energy that it can absorb during the crushing process. For that reason and in order to estimate the effect of the damage size on this important panel property, the energy absorption of the intact, damaged and tube-reinforcement damaged panels was determined from the numerically obtained stress–strain curve using the Trapz function in Matlab^®^ (Equation (11)). In an effort to better estimate the loss of energy absorption capacity, the relative energy absorption efficiency (REAE) was introduced. This parameter is defined as the ratio between the absorbed energy by the damaged panels (or absorbed energy by the tube-reinforced damaged panels in case of reinforcement tubes applications) and the absorbed energy by the intact ones. Details of absorbed energy of intact and damaged panel configurations are summarized in Table 3. It was found that the absorbed energy significantly decreases as the damage size increases (20% damaged panel loses approximately 50% of the energy absorption capacity compared to intact ones).

### 4.3. Tube-Reinforced Damaged Panel Models

**Figure 17:**
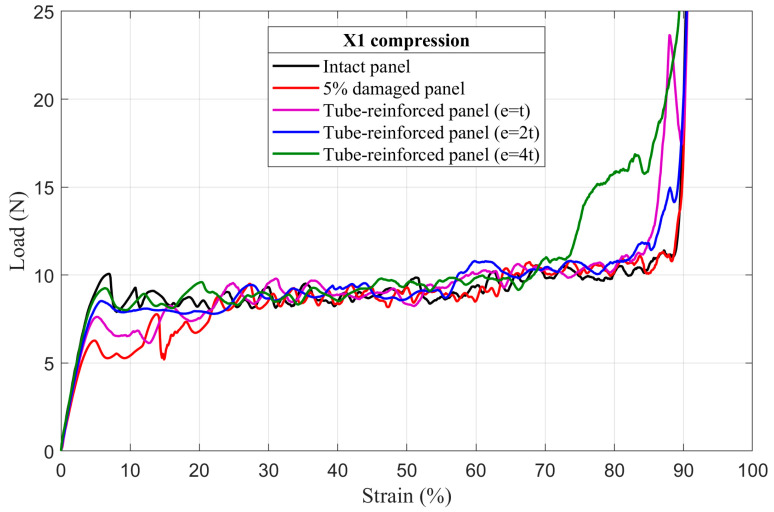
The load–strain behavior of tube-reinforced 5% damaged panels under compression in the X_1_ direction.

**Figure 18:**
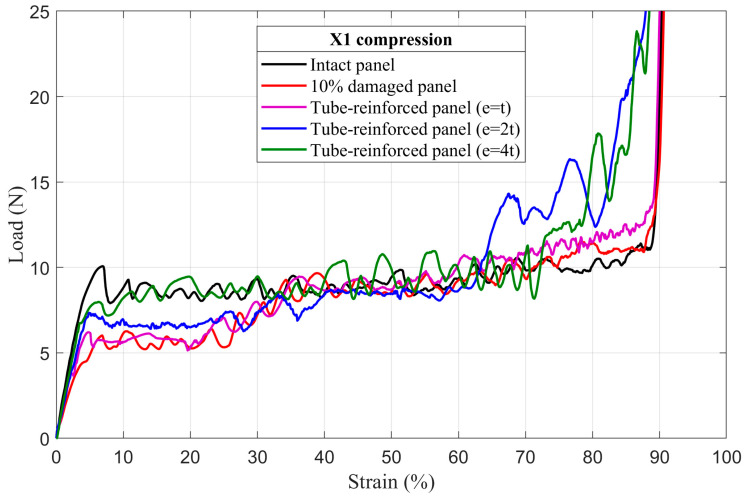
The load–strain behavior of tube-reinforced 10% damaged panels under compression in the X_1_ direction.

**Figure 19:**
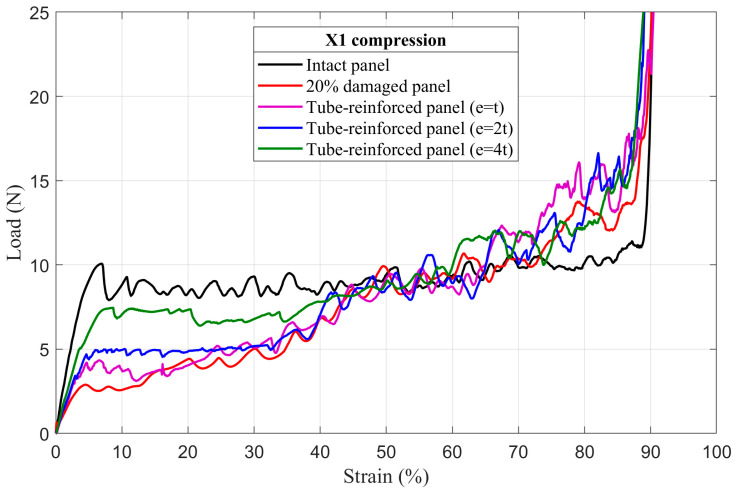
The load–strain behavior of tube-reinforced 20% damaged panels under compression in the X_1_ direction.

**Figure 20:**
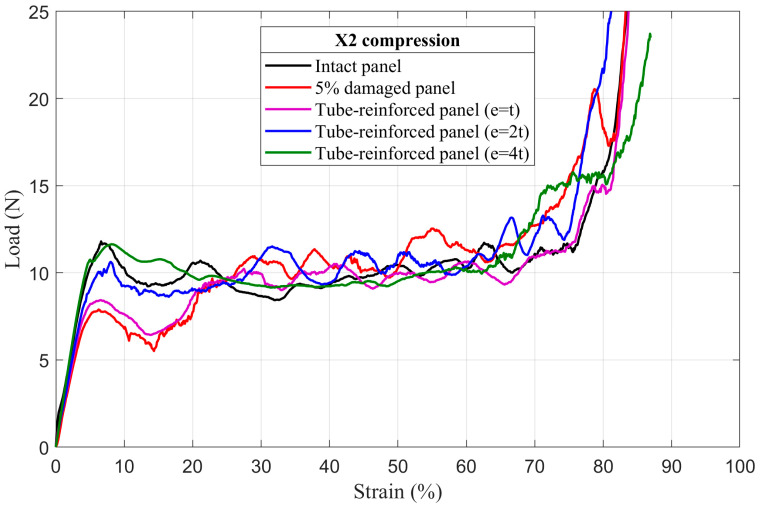
The load–strain behavior of tube-reinforced 5% damaged panels under compression in the X_2_ direction.

**Figure 21:**
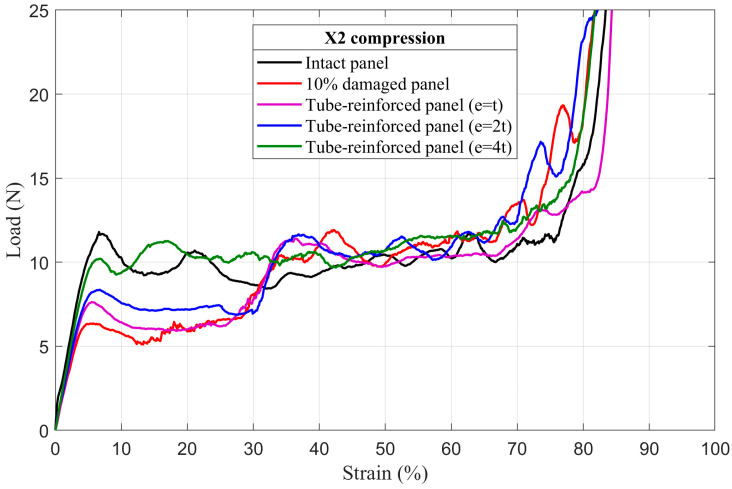
The load–strain behavior of tube-reinforced 10% damaged panels under compression in the X_2_ direction.

**Figure 22:**
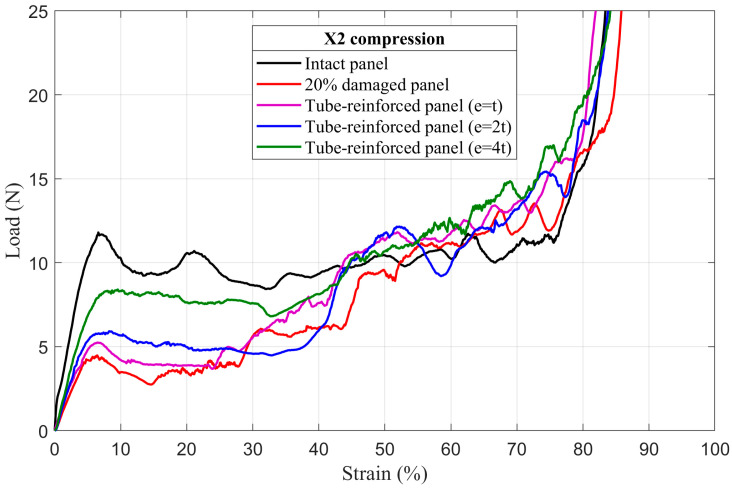
The load–strain behavior of tube-reinforced 20% damaged panels under compression in the X_2_ direction.

In order to investigate the improvements obtained by inserting the reinforcement tubes with three different wall thicknesses *e* (*t*; *2t*; *4t*) into the cut hole of the damaged panels, tube-reinforced damaged panels are also subjected to compressive loading along the X_1_ and X_2_ directions. To facilitate the comparison of the results, intact, damaged, and tube-reinforced damaged panels with different tube thicknesses are represented in the same load–strain curve for all of the damage sizes (5%, 10%, and 20%), Figure 17, Figure 18, Figure 19, Figure 20, Figure 21 and Figure 22.

#### 4.3.1. First Maximum Compressive Load

It can be observed that the first maximum compressive load of all damaged panels, in both loading directions, was increased by inserting the reinforcement tubes regardless of their thickness. These results are a direct consequence of the action of the tube which takes over the role of supporting the cut cell walls within the damage zone and provide a larger contact surface reducing the stress concentration, Figure 17, Figure 18, Figure 19, Figure 20, Figure 21 and Figure 22. The thickness of the reinforcement tubes has an important impact on the crashworthiness performance of damaged panels. In the case of small damage size (5% damage), the first maximum compressive load can be restored using the tubes with thicknesses *e* = *2t* and *e* = *4t*. On the other hand, the tube with thickness *e* = *t* slightly increases this parameter. In the same way, the tube with thickness *e* = *4t* shows significant improvement in the panels with greater damage size (10% and 20%). However, the reinforcement tube with *e* = *2t* provides a slight increase in the $\sigma_{\max}^{*}$ for the 10% damaged panels but not for the 20% damaged panels.

#### 4.3.2. Deformation Mode

**Figure 23:**
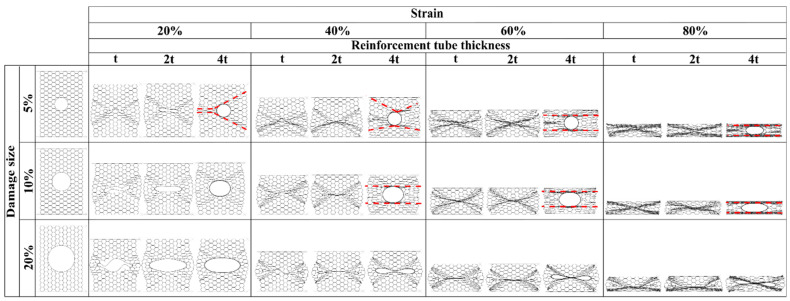
Sequence of deformed configurations of tube-reinforced damaged panels under compression in the X_1_ direction.

**Figure 24:**
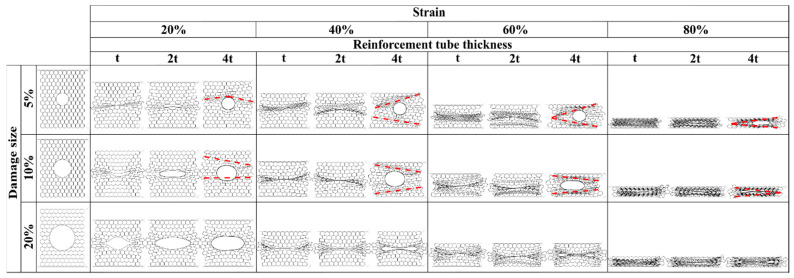
Sequence of deformed configurations of tube-reinforced damaged panels under compression in the X_2_ direction.

The deformation modes of the tube-reinforced damaged panels are in the most cases like the intact ones according to the loading direction. In case of compression along the X_1_ loading direction of the 5% and 10% damaged panels, the reinforcement using the tube of thickness *e* = *4t* produced a sort of disorder in the deformation mode due to the excessive stiffness of the tube compared to the honeycomb panels. In this case, deformation mode is characterized by piling up of the honeycomb rows above and below the tube until the entire collapse of the panels. After that, the tubes began to fold, Figure 23. On the other hand, along X_2_ loading direction, the tubes slide to the right or the left side from the middle of the panel. As consequence, the “I” band was not perpendicular to the load but showed a slope, Figure 24.

#### 4.3.3. Mean Crushing Load

For the 5% damaged panels, the reinforcement tube with a thickness of *e* = *t* cannot provide enough resistance against crushing, so it directly folds, and the panel behave as the damaged one. On the other side, the reinforcement tubes with *e* = *2t* and *e* = *4t* are sufficiently stiff to fold gradually and stay in contact with the cut cell walls in the damaged zone during the collapse. Consequently, the mean crushing load can be restored and the staircase effect vanished for these reinforced-damaged panels, Figure 17 and Figure 20.

When the damage increases, the tubes must have a greater thickness to be stiffer. In case of the 10% damaged panels, only tubes with *e* = *4t* could restore the mean crushing load and reduce the staircase effect due to the damage, Figure 18 and Figure 21. For the 20% damaged panels, none of the three tube thicknesses could restore the mean crushing load due to the significant level of damage size, as shown in Figure 19 and Figure 22.

#### 4.3.4. Energy Absorption

The insertion of the reinforcement tubes in the damaged panels could reinstitute, in both X_1_ and X_2_ loading directions, their relative energy absorption efficiency, as shown in Table 3. However, it should be noted that the reinforcement tube with thickness *e* = *t* slightly increases this parameter compared to the improvement achieved by the one with the thicknesses *e* = *2t* and *e* = *4t*. Nevertheless, the energy absorption capacity in the case of the 5% and 10% damaged panels can be completely recovered using the reinforcement tube thickness *e* = *4t*. These results are in direct relationship with the fold mechanism of the reinforcement tube explained in previous subsection.

## 5. Conclusions

The main objective of presented numerical simulation of in-plane crushing of intact, damaged, and tube-reinforced damaged 5052 aerospace grade aluminum alloy honeycomb was to investigate the degradation of honeycomb panel characteristics as well as the possibility of improving it using reinforcement tubes with different thicknesses.

In order to validate the FE model of intact panel, it was submitted to uniaxial quasi-static compressive loading simulation test in X_1_ and X_2_ directions. It was found that the simulation results are in very good agreement with the theoretical values and the experimental results [18]. Therefore, it has clearly validated that the FE model of intact panel is able to predict the crushing performance of damaged and tube-reinforced damaged honeycomb panels. The in-plane crushing behaviors and energy absorption capacities of the intact, damaged, and tube-reinforced damaged panels with different damage sizes in both directions, are numerically simulated by using the nonlinear FE method Abaqus/Explicit, and the crashworthiness performances are compared with each other. With respect to the research it is possible to draw the following conclusions:

- The first maximum compressive load and the mean crushing load of damaged panels decreases as the damage size increases.
- The plateau regimes of all damaged panels are characterized by a staircase increase in load due to the gradual closure of the damage hole.
- The energy absorption capacity of the damaged panels significantly decreases as the damage size increases and, in the case of 20% damaged panels, reach the approximatively 50% of the intact panel capacity.
- The first maximum compressive load of all damaged panels, in both loading directions, can be increased by inserting the reinforcement tubes regardless of their thickness. The increase in maximum compressive load is directly proportional to the thickness of the reinforcement tube.
- The mean crushing load can be completely recovered, and the staircase effect in plateau regime vanished, for the 5% damaged panels by using the reinforcement tube with the thicknesses *e* = *2t* and *e* = *4t*. In case of the 10% damaged panels, only tubes with *e* = *4t* can restore the mean crushing load and reduce the staircase effect. For the 20% damaged panels, none of the three tube thicknesses can restore this parameter.
- The relative energy absorption efficiency of the damaged panels can be enhanced by inserting the reinforcement tubes, especially in the case of the 5% and 10% damaged panels for which the entire energy absorption capacity was recovered with the reinforcement tube thickness *e* = *4t*.

## Figures and Tables

**Table 1:** Finite element model mesh parameters.

Component	Element Size (mm)	Element Type	Number of Elements		Number of Nodes	
Honeycomb intact panel	0.4	S4R	**X_1_**	14,868	**X_1_**	21,825
			**X_2_**	13,384	**X_2_**	19,650
Top rigid plate	0.4	R3D4	**X_1_**	550	**X_1_**	828
			**X_2_**	550	**X_2_**	828
Bottom rigid plate	0.4	R3D4	**X_1_**	550	**X_1_**	828
			**X_2_**	550	**X_2_**	828
Honeycomb panel (5% damage)	0.4	S4R	**X_1_**	14,168	**X_1_**	20,814
			**X_2_**	12,760	**X_2_**	18,750
Honeycomb panel (10% damage)	0.4	S4R	**X_1_**	13,424	**X_1_**	19,734
			**X_2_**	12,232	**X_2_**	17,976
Honeycomb panel (20% damage)	0.4	S4R	**X_1_**	11,960	**X_1_**	17,604
			**X_2_**	10,936	**X_2_**	16,104
Reinforcement tube (5% damaged panel)	0.4	S4R	**X_1_**	458	**X_1_**	687
			**X_2_**	436	**X_2_**	654
Reinforcement tube (10% damaged panel)	0.4	S4R	**X_1_**	648	**X_1_**	972
			**X_2_**	616	**X_2_**	924
Reinforcement tube (20% damaged panel)	0.4	S4R	**X_1_**	916	**X_1_**	1374
			**X_2_**	870	**X_2_**	1305

**Table 2:** Results of intact panel compression in X_1_ and X_2_ directions.

Intact Panel			Young’s Modulus (MPa)	First Maximum Compressive Strength (MPa)	Plateau Stress (MPa)
**compression direction**	**X_1_**	Numerical results	2.693	0.132	0.126
		Theoretical values	2.908	-	0.135
	**X_2_**	Numerical results	2.654	0.147	0.132
		Theoretical values	2.908	-	0.135
		Experimental results [18]	2.178	0.131	0.118

**Table 3:** Energy absorption of intact, damaged and tube-reinforced damaged panels.

Configuration			Intact Panel		Damaged Panel		Tube-Reinforced Damaged Panel					
							*e*					
							*t*		*2t*		*4t*	
			EA(J)	REAE(%)	EA(J)	REAE(%)	EA(J)	REAE(%)	EA(J)	REAE(%)	EA(J)	REAE(%)
**Compression Direction**	**X_1_**	**Intact panel**	0.599	100.0								
		5% damaged panel			0.532	88.8	0.565	94.3	0.581	97.0	0.605	101.0
		10% damaged panel			0.476	79.5	0.493	82.3	0.505	84.3	0.597	99.7
		20% damaged panel			0.335	55.9	0.340	56.8	0.373	62.3	0.473	79.0
	**X_2_**	**Intact panel**	0.585	100.0								
		5% damaged panel			0.544	93.0	0.537	92.0	0.591	101.0	0.594	101.5
		10% damaged panel			0.478	81.7	0.491	84.0	0.523	89.4	0.616	105.3
		20% damaged panel			0.304	52.0	0.364	62.2	0.382	65.3	0.487	83.2

## Data Availability

The data presented in this study are available on request from the corresponding author.

## Nomenclature

$E_{1}^{*}$	Young’s modulus of honeycomb panel in X_1_ direction, N/mm^2^
$E_{2}^{*}$	Young’s modulus of honeycomb panel in X_2_ direction, N/mm^2^
*E*_S_	Young’s modulus of honeycomb cell wall material, N/mm^2^
*E**	Young’s modulus of regular hexagonal honeycomb panel, N/mm^2^
*E*_tan_	tangent modulus of honeycomb cell wall material, N/mm^2^
*t*	cell wall thickness, mm
*l*	length of single cell wall, mm
*h*	length of double cell wall, mm
*b*	honeycomb panel thickness, mm
*θ*	internal angle of the cell walls, degree
$\nu_{12}^{*}$	Poisson’s ratio of honeycomb panel in X_1_ direction
$\nu_{21}^{*}$	Poisson’s ratio of honeycomb panel in X_2_ direction
*ν**	Poisson’s ratio of regular hexagonal honeycomb panel
*ν*	Poisson’s ratio of honeycomb cell wall material
$G_{12}^{*}$	Shear modulus of honeycomb panel in X_1_–X_2_ plane, N/mm^2^
${\left(\sigma_{pl}^{*}\right)}_{1}$	plateau stress of honeycomb panel in X_1_ direction, N/mm^2^
${\left(\sigma_{pl}^{*}\right)}_{2}$	plateau stress of honeycomb panel in X_2_ direction, N/mm^2^
$\sigma_{pl}^{*}$	plateau stress of regular hexagonal honeycomb panel, N/mm^2^
$\sigma_{ys}^{*}$	yield strength of honeycomb cell wall material, N/mm^2^
$\sigma_{\max}^{*}$	first maximum compressive strength of honeycomb panel, N/mm^2^
$\sigma_{130}^{*}$	plateau end (1.3 times the plateau stress), N/mm^2^
*EA*	energy absorption of honeycomb panel, J
*REAE*	relative energy absorption efficiency, %
*L* ^0^	original length of honeycomb panel, mm
*F*	force (load), N
*ε*	strain
*ε*_50%_	50% strain
*ε*_ple_	plateau end strain
*e*	reinforcement tube thickness, mm
*ρ*	density of honeycomb cell wall material, g/cm^3^
*V*	velocity of moving top rigid plate, mm/s
*f*	frictional coefficient
